# Feasibility and Preliminary Educational Outcomes of Self-Reported Clinical Confidence After a Global Health Experience: A Pilot Study

**DOI:** 10.7759/cureus.116842

**Published:** 2026-09-24

**Authors:** Micah J Thomas, Madison K Sobarzo, Samuel A Sobarzo, David Oancea, Paul Oancea, Joyce P Hnatek

**Affiliations:** 1 Primary Care and Rural Medicine, Texas A&M University College of Medicine, Houston, USA; 2 Primary Care and Rural Medicine, Texas A&M University College of Medicine, Dallas, USA; 3 Primary Care and Rural Medicine, Texas A&M University College of Medicine, College Station, USA

**Keywords:** clinical skills training, global health, medical education, medical spanish, preclinical medical students, self-efficacy, short-term medical missions

## Abstract

Background

Short-term international medical mission trips are a ubiquitous but understudied component of preclinical medical education, especially in the United States. Self-efficacy -- a trainee’s belief in their own capacity to execute a clinical task -- is theorized to precede and shape skill acquisition. However, item-level changes in self-reported clinical confidence surrounding a brief, immersive mission trip, particularly among students without prior fluency in the trip’s local language, have not been well characterized.

Methods

Preclinical (M1), non-Spanish-speaking students from a Christian Medical Association chapter completed an investigator-developed, 10-item clinical confidence questionnaire (5-point Likert scale) before and after a one-week, pop-up, free clinic mission trip to Lima, Peru (10-14 March 2025). The questionnaire has not been formally validated. Ten students completed the pre-trip survey, seven completed the post-trip survey, and five completed both and formed the matched analytic sample. Paired t-tests, Wilcoxon signed-rank tests, and paired Cohen’s d were used to explore pre-to-post-trip change, with inferential results interpreted cautiously because of the very small matched sample.

Results

Among the five matched students, composite self-reported clinical confidence increased from a mean of 28.8 pre-trip to 43.0 post-trip, a 49% relative increase (Cohen’s d = 3.08; Wilcoxon p = 0.063). The largest observed increases were in confidence during a foreign-language patient encounter (1.4 to 4.0, d = 4.75) and generating treatment recommendations (1.4 to 3.8, d = 4.38). The smallest observed increases were in skills already rated highly at baseline, including communicating with a patient (4.2 to 4.4) and taking vital signs (4.2 to 4.6).

Conclusions

In this pilot cohort, higher post-trip scores were observed across several self-reported clinical confidence domains. However, 50% attrition from the pre-trip survey to the matched analytic sample, absence of a comparison group, use of an investigator-developed and unvalidated questionnaire, and variable post-trip survey timing (13-24 days after return) substantially limit inference. These findings are hypothesis-generating and describe changes in self-reported confidence only; they should not be interpreted as evidence of improved objective clinical competence.

## Introduction

Self-efficacy is a trainee’s belief in their own capacity to successfully execute a task, and it is theorized to shape subsequent motivation, persistence, and skill acquisition [1]. In medical education specifically, self-efficacy for clinical skills is thought to develop through direct performance experience, observation of others, and feedback [2]. Preclinical students who report higher clinical self-efficacy are generally more engaged and higher-achieving learners [3,4]. Because self-efficacy is domain-specific rather than a single global trait, understanding how self-reported confidence varies across skill domains surrounding a given educational experience requires measurement at the level of individual skills rather than a single aggregate score [1,3,5].

Short-term global health outreach experiences (GHOEs), otherwise referred to as medical mission trips, are an almost universal extracurricular offering in U.S. medical schools which, in contrast to international medical clerkship rotations, are undertaken at the students’ expense and commonly without any corresponding class credit [6-9]. However, only a small body of literature has examined changes in trainees’ clinical confidence surrounding GHOEs, with much of this work focused on pre-departure curricula and simulations [10]. Pilot studies of pre-departure simulation curricula have reported higher confidence in basic clinical tasks such as taking vital signs and conducting an initial patient interview after brief, structured training sessions [11,12]. There is a paucity of literature that explores these confidence changes by bookending the live trip experience itself with a true pre- and post-trip measurement.

Language-concordant care is an important and growing need in the U.S. medical curriculum [13,14]. Spanish is the fastest-growing non-English language spoken by patients nationally, yet fewer than a quarter of U.S. medical schools’ Spanish-language programs meet basic curricular standards for structure, assessment, and course credit, making the gap between need and education very wide [15,16]. Most directly relevant to our study, a previous cohort study of a U.S.-based, Spanish-speaking medical student service trip found that among several confidence domains measured before and after the trip, confidence communicating in Spanish showed the largest post-trip increase [17]. That study’s cohort was not stratified by medical student year, included Spanish-speaking participants, and did not examine whether medical Spanish-speaking confidence changed proportionally with other skill confidence. Whether a similar pattern is observed among non-Spanish-speaking first-year students, across a broader panel of clinical skill domains, has not yet been reported at the item level.

In contrast to the relative paucity of language-concordant care training in medical education, clinical skills such as history-taking, physical examination, and vital-sign measurement are, justifiably, a core focus of most preclinical curricula. They are typically taught and reinforced through Objective Structured Clinical Examination (OSCE)-linked coursework. Near-peer and simulation-based basic-skills programs have been associated with higher preclinical students’ self-assessed confidence in these same domains before any GHOE or elective occurs [18]. It is therefore important to determine whether the pattern of self-reported confidence change surrounding a short-term GHOE differs between skills a standard curriculum already targets and skills that receive less emphasis, a distinction prior GHOE-confidence pilots have not examined at the item level.

We report pilot data from a one-week, Christian Medical Association (CMA)-organized pop-up, free clinic trip to Lima, Peru, to characterize pre-to-post-trip changes in a 10-item, domain-specific clinical confidence panel and generate hypotheses among first-year preclinical, non-Spanish-speaking medical students at Texas A&M University College of Medicine.

## Materials and methods

Setting and participants

This study was conducted among first-year (M1) medical students affiliated with a CMA student chapter at Texas A&M University College of Medicine who participated in a one-week pop-up free clinic mission trip to Lima, Peru, from 10 to 14 March 2025. Participation in the trip was voluntary and self-selected; no non-traveling comparison group was surveyed for the confidence outcome. Ten students completed a pre-trip clinical confidence questionnaire, and seven completed an identical post-trip questionnaire; five students completed both forms and serve as the matched pilot sample. Pre-trip responses were completed 2-7 days before departure, and completed post-trip responses were received 13-24 days after return. During the trip, first-year students were paired with second- or fourth-year counterparts for clinical encounters. Spanish interpreters were used by each medical student pair to facilitate communication. The student pairs conducted the clinical encounter and then presented their findings to an attending physician, who re-interviewed the patient as needed and approved the plan and prescriptions. The student-to-teacher ratio was approximately 2:1. Approximately 340 patients were seen over 5 clinic days.

Instruments

Clinical confidence was assessed with a 10-item, investigator-developed questionnaire administered via an online form. The instrument is exploratory and has not undergone formal psychometric validation. It was developed by the trip organizers to map onto commonly cited preclinical skill domains and was informed by item content used in prior GHOE confidence pilots as well as Texas A&M University College of Medicine internal curricular quality-improvement surveys [11,12,17]. Items asked how confident respondents felt 1) communicating with a patient, 2) taking vital signs, 3) taking a comprehensive history, 4) performing a comprehensive physical exam, 5) taking a focused history, 6) performing a focused physical exam, 7) generating differential diagnoses, 8) generating treatment recommendations, 9) presenting a case to an attending, and 10) engaging in a patient encounter in a foreign language. Each item used a 5-point ordinal scale (1 = not confident at all, 2 = slightly confident, 3 = somewhat confident, 4 = fairly confident, 5 = very confident), which we treated as approximately interval for the purpose of computing means, paired-sample statistics, and a composite score (sum of the 10 items; range 10-50). The instrument is reproduced in the Appendices. Although its original title uses the term “competency,” the items assessed self-reported confidence and not objective clinical competence.

Data linkage and statistical analysis

Confidence survey responses were matched pre-to-post by institutional email address. Ordinal responses were coded 1-5 as above. Pre-to-post changes were summarized with mean differences and paired Cohen’s d, calculated as the mean difference divided by the standard deviation of the differences. Paired t-tests were calculated as an exploratory parametric analysis, and Wilcoxon signed-rank tests were used as a nonparametric sensitivity analysis. Given the matched sample of n = 5, inferential p-values were treated as exploratory and were not used as confirmatory evidence. For the composite score, all five paired differences were nonzero; with n = 5, the minimum attainable two-sided exact Wilcoxon p-value is 0.0625. All analyses were conducted in Python (pandas, SciPy).

Ethical considerations

This study was reviewed by the Division of Research, Human Subjects Research Protection Program (HRPP) at Texas A&M University under the exemption number STUDY2025-0975. The HRPP determined on 24 November 2025 that this research meets the criteria for Exemption in accordance with 45 CFR 46.104. Surveys were initially only administered for internal use, but the desire to publish anonymized results led to the application for IRB exemption.

## Results

Table 1 summarizes survey participation and the matched analytic sample. The five matched students were all M1s (four male, one female). Of the 10 students who completed the pre-trip survey, five had a matched post-trip response and were included in the paired analysis, corresponding to 50% attrition from pre-trip participation to the matched analytic sample. 

**Table 1:** Survey participation and analytic sample

Survey	Respondents (n)	Timing relative to trip
Pre-trip clinical confidence survey	10	2–7 days before departure
Post-trip clinical confidence survey	7	13–24 days after return
Matched pre–post pairs (analytic sample)	5	—
Completed pre-trip survey only (excluded)	5	—
Completed post-trip survey only (excluded)	2	—

Clinical confidence

Composite self-reported clinical confidence increased from a mean of 28.8 (range 20-35) before the trip to 43.0 (range 34-47) after the trip, an observed mean difference of 14.2 points and a 49% relative increase (Cohen’s d = 3.08). The exact Wilcoxon signed-rank test yielded p = 0.063. Given the very small sample and limited exact-test resolution, inferential p-values are reported for transparency rather than interpreted as confirmatory. Each of the five matched students improved on every item on which they had not already rated themselves at ceiling before the trip (Figure 1).

**Figure 1:**
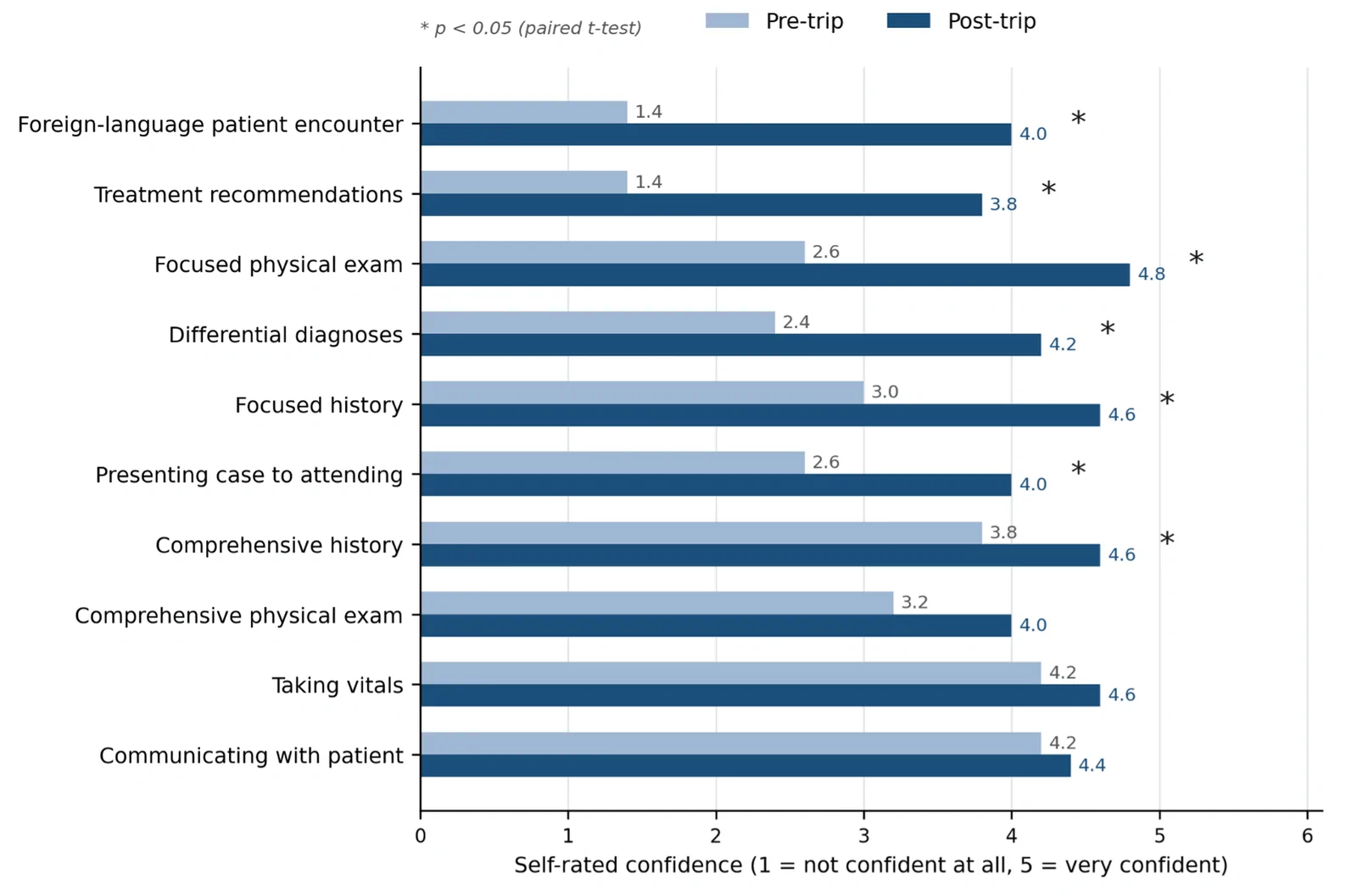
Pre- vs post-trip clinical confidence by skill domain Pre- and post-trip self-rated clinical confidence, sorted by magnitude of gain (n = 5 matched students). * p < 0.05, paired t-test

Observed changes were not uniform across domains. In respective order, the largest increases were observed in confidence engaging in a patient encounter in a foreign language (1.4 to 4.0; +2.6; d = 4.75), generating treatment recommendations (1.4 to 3.8; +2.4; d = 4.38), performing a focused physical exam (2.6 to 4.8; +2.2; d = 2.63), generating differential diagnoses (2.4 to 4.2; +1.8; d = 2.15), taking a focused history (3.0 to 4.6; +1.6; d = 2.92), and orally presenting a case to an attending (2.6 to 4.0; +1.4; d = 2.56). The smallest observed increases were in domains already rated relatively highly before the trip: communicating with a patient (4.2 to 4.4; +0.2; d = 0.45) and taking vital signs (4.2 to 4.6; +0.4; d = 0.73) (Table 2).

**Table 2:** Pre- and post-trip clinical confidence by skill domain (n = 5 matched students; 5-point scale) Δ, mean post-trip minus mean pre-trip score Improved/same/worse counts the number of the five matched students whose individual score increased, was unchanged, or decreased.

Skill domain	Pre	Post	Δ	Improved/same/worse	Paired t p	Wilcoxon p	Cohen’s d
Foreign-language patient encounter	1.4	4	2.6	5 / 0 / 0	<0.001	0.063	4.75
Treatment recommendations	1.4	3.8	2.4	5 / 0 / 0	0.001	0.063	4.38
Focused physical exam	2.6	4.8	2.2	5 / 0 / 0	0.004	0.063	2.63
Differential diagnoses	2.4	4.2	1.8	5 / 0 / 0	0.009	0.063	2.15
Focused history	3	4.6	1.6	5 / 0 / 0	0.003	0.063	2.92
Presenting case to attending	2.6	4	1.4	5 / 0 / 0	0.005	0.063	2.56
Comprehensive history	3.8	4.6	0.8	4 / 1 / 0	0.016	0.125	1.79
Comprehensive physical exam	3.2	4	0.8	2 / 3 / 0	0.242	0.5	0.61
Taking vitals	4.2	4.6	0.4	2 / 3 / 0	0.178	0.5	0.73
Communicating with patient	4.2	4.4	0.2	1 / 4 / 0	0.374	1	0.45
Composite score (sum of 10 items, range 10–50)	28.8	43	14.2	5 / 0 / 0	0.002	0.063	3.08

## Discussion

In this hypothesis-generating cohort, the five first-year medical students who completed both a pre- and post-trip confidence survey reported large observed increases in self-reported clinical confidence following the GHOE. The composite score increased from 28.8 to 43.0, with a large paired effect size (d = 3.08); however, the exact Wilcoxon test yielded p = 0.063, and the matched sample was too small for stable inferential conclusions. The direction of change is consistent with prior reports of higher self-reported confidence after short-term global health experiences or related pre-departure training [10,11,12,17]. However, because this study had no comparison group and post-trip responses were collected 13-24 days after return, the observed changes cannot be attributed specifically to the trip. Maturation, ongoing preclinical coursework, recall effects, or other time-varying factors may also have contributed.

The clinical setting itself remains important context. Students participated in supervised, high-volume patient care in an unfamiliar environment, with interpreters available for each student pair. Student pairs presented their findings to an attending physician, and attendings re-interviewed patients as needed before approving plans and prescriptions. The approximately 2:1 student-to-teacher ratio allowed close supervision. This supervision is an important part of keeping short-term global health experiences safe and educational, but this study did not objectively measure whether students’ clinical performance or competence changed.

The item-level pattern is also informative. Observed changes were smallest in domains that students already rated relatively highly before departure: communicating with a patient, performing a comprehensive physical exam, and taking vital signs. These are core preclinical skills practiced repeatedly in OSCE-linked coursework. Prior basic-skills programs have similarly reported relatively high self-assessed confidence in these domains before any mission trip or elective occurs [18]. The small observed changes in these areas may therefore reflect a ceiling effect, although the current sample is too small to establish that explanation.

In contrast, the largest observed changes occurred in domains that either reflect later-stage clinical reasoning tasks, such as differential diagnosis, treatment recommendations, and oral case presentation, or were specific to the trip’s linguistic context, particularly the foreign-language patient encounter. This pattern parallels a prior Spanish-speaking service-trip cohort in which confidence communicating in Spanish showed the largest post-trip change among measured confidence domains [17]. Our results extend that observation descriptively to an exclusively non-Spanish-speaking first-year cohort, but they do not establish that the trip caused the change or that objective skill improved. Instead, they support the hypothesis that foreign-language communication and higher-order clinical reasoning may be useful domains to examine prospectively in larger, controlled studies. Given that many U.S. medical Spanish programs remain limited in structure, assessment, and course credit [15,16], it is also reasonable to study whether immersive experiences complement formal medical Spanish curricula; that question requires validated measures and objective performance outcomes.

Limitations

A major limitation is attrition. Although 10 students completed the pre-trip survey, only 5 had matched post-trip responses and were included in the paired analysis, resulting in 50% attrition from pre-trip participation to the matched analytic sample. Nonresponders may have experienced smaller, no, or different changes in confidence than responders. If so, the observed paired changes could overestimate or otherwise misrepresent the experience of the original pre-trip cohort, introducing both attrition and response bias.

The paired analytic sample was also extremely small (n = 5), with all participants drawn from a single trip at a single institution, substantially limiting statistical power, precision, and generalizability [19]. Inferential findings should therefore be interpreted as exploratory rather than confirmatory. Trip participation was voluntary and self-selected, and no non-traveling comparison group was included. Consequently, changes associated with maturation, concurrent preclinical coursework, or other time-varying factors cannot be separated from changes observed following the global health experience.

Post-trip survey timing varied from 13 to 24 days after return. This variation may itself have introduced heterogeneity in recall and response, and students continued their regular medical education during the interval. Intervening coursework or clinical learning therefore represents a potential confounder when interpreting the post-trip scores.

The confidence instrument was investigator-developed and has not undergone formal psychometric validation. Self-reported confidence does not necessarily correspond to clinical competence and may also be susceptible to response-shift bias, whereby direct clinical exposure alters participants’ internal standards for judging their own confidence [20-22]. No objective assessment of clinical performance or competence was included. The paired sample was also gender-imbalanced (four male and one female participant), precluding meaningful subgroup analysis. Participants were not matched according to prior GHOE experience or specialty interest, which may have influenced baseline confidence or responses to the experience.

Finally, because participation was self-funded, socioeconomic factors may have contributed to selection bias. The survey was also distributed by student trip organizers, and social desirability bias therefore cannot be excluded.

Future directions

Given the annual nature of this trip at the authors’ home institution, this pilot study identified several concrete directions for future investigation. First, future cohorts should use strategies to reduce follow-up attrition and a prespecified, narrow post-trip survey window. A parallel comparison group of students who do not participate in a GHOE but are evaluated over the same academic interval would help distinguish trip-associated changes from maturation and concurrent curricular exposure. Second, future evaluations should incorporate validated confidence instruments and objective measures of clinical competence, such as OSCEs or standardized patient encounters, alongside self-reported confidence so that perceived and demonstrated skill can be evaluated separately. Third, larger samples of first-year medical students would permit adjustment for prior global health experience and other participant characteristics that may influence educational outcomes. Finally, questions about how participation is associated with later specialty choice and preferences could be explored in future investigations.

## Conclusions

Among five first-year medical students with matched surveys, self-reported clinical confidence was higher after a one-week, CMA-organized mission trip to Lima, Peru, with the largest observed changes in foreign-language communication and higher-order clinical reasoning domains rather than basic skills rated highly before departure. However, the very small matched sample, 50% attrition, lack of a comparison group, investigator-developed and unvalidated questionnaire, and variable 13-24-day timing of post-trip responses substantially limit interpretation. These findings are best treated as hypothesis-generating. They describe changes in self-reported confidence and should not be interpreted as evidence of improved objective clinical competence. Future work should pair validated confidence measures with objective performance assessments, tighter survey timing, improved follow-up, and a non-traveling comparison group.

## Appendices

Medical competency self-assessment survey

The following instrument was adapted from a Google Form format for publication.

Part 1. Anonymous Demographics

1. Are you a first-year medical student (M1)? (Yes / No)

2. What is your gender? (Male / Female)

Part 2. Clinical Competency Self-Assessment

Respondents were asked to rate their level of confidence in performing each of the following clinical tasks, using the scale below: 1 = Not confident at all, 2 = Slightly confident, 3 = Somewhat confident, 4 = Fairly confident, 5 = Very confident.

**Table 3:** Clinical competency self-assessment survey items and confidence rating scale Note: Each item was presented as a single-select (radio-button) question with the following response options: 1 = Not confident at all; 2 = Slightly confident; 3 = Somewhat confident; 4 = Fairly confident; 5 = Very confident.

No.	Clinical Skill	1	2	3	4	5
1	Communicating with a patient					
2	Taking vital signs on a patient					
3	Taking a comprehensive patient history					
4	Performing a comprehensive physical exam					
5	Taking a focused history					
6	Performing a focused physical exam					
7	Creating differential diagnoses for common patient complaints					
8	Creating treatment recommendations for common patient complaints					
9	Presenting a patient's case to an attending physician					
10	Engaging in a patient encounter in a foreign language					
